# Rethinking psychometric testing in autism: overcoming the challenges of comorbidity and diagnostic overshadowing

**DOI:** 10.1017/S1092852925000021

**Published:** 2025-01-17

**Authors:** Nihit Gupta, Srinija Srinivasan, Mayank Gupta

**Affiliations:** 1 Department of Child and Adolescent Psychiatry, Dayton Children’s Hospital, Dayton, OH, USA; 2 Northwestern University, Chicago, IL, USA; 3 Southwood Children Behavioral Health, Pittsburgh, PA, USA

**Keywords:** autism, ados, diagnosis, disparity, barriers in care, misdiagnosis

## Abstract

Epidemiological data indicates a rising prevalence of autism spectrum disorder (ASD) among eight-year-old children, with rates increasing from 1 in 44 to 1 in 36 between 2022 and 2023. This growing prevalence poses significant challenges in achieving accurate diagnoses, particularly due to comorbid conditions and diagnostic overshadowing. Certain subgroups—such as females with ASD, individuals with high cognitive abilities, and ethnic minorities—remain at heightened risk of underdiagnosis. Diagnostic tools like the Autism Diagnostic Observation Schedule (ADOS-2) have limitations, particularly in clinical settings where gender biases and cultural differences in symptom presentation can complicate accurate assessment. Moreover, rural areas face additional burdens due to limited access to care, further exacerbating diagnostic challenges. The review underscores the necessity for improved screening and diagnostic methods tailored to diverse populations, acknowledging the current limitations of existing tools. It also highlights significant barriers such as workforce shortages and lengthy wait times for evaluations. Emphasizing the importance of clinician education and targeted diagnostic approaches, the review calls for attention to cultural and gender differences in ASD evaluation.

## Introduction

On March 24, 2023, the Morbidity and Mortality Weekly Report from the Center for Disease Control and Prevention revealed a concerning trend in the prevalence of autism spectrum disorder (ASD). The newer estimates, utilizing data from 2020 collected across 11 Autism and Developmental Disabilities Monitoring Network (ADDM Network) sites nationwide, indicate a rise from 1 in 44 for children of 8 years of age in 2022 to 1 in 36 in 2023 ^1^ in the United States.

The increasing prevalence of ASD can be attributed to several factors, including heightened awareness among clinicians and families, and the evolution of diagnostic criteria in successive DSM editions. This trend is not surprising to clinicians actively engaged in practice, as there has been an accretion of milder forms of ASD diagnoses with the advent of widely used screening and diagnostic tools like ADOS-2 and ADI-R. Additionally, the growing recognition of undiagnosed and misdiagnosed ASD cases within clinical samples has played a role. Improved epidemiological data collection now includes low-income, rural, ethnic minority, and other marginalized groups that have traditionally faced limited access to mental health services, further contributing to the observed increase in prevalence.^2^

The absence of timely access to ASD evaluations, including but not limited to workforce shortages, is a prominent issue resulting in extended wait times for assessments. A staggering 61% of organizations reported wait times exceeding 4 months, with 15% experiencing delays of over a year for psychiatric evaluations.^3^

The prevalence of ASD in clinical samples is reported to be 5 times higher than in the general community. A study revealed that 1 in 10 adults admitted to inpatient facilities had a history of suicidal attempts with possible underlying ASD symptoms.^4^ The escalating burden of undiagnosed ASD carries profound implications at both individual and systemic levels. Individuals with ASD face an elevated likelihood of emergency room visits, often presenting with debilitating cooccurring conditions, and are at a higher risk of death by suicide.^5^ These findings underscore the urgent need for increased awareness of the signs and symptoms of ASD, timely access to evaluations, and enhanced mental health support for individuals on the autism spectrum.

## Method

**Figure 1.**
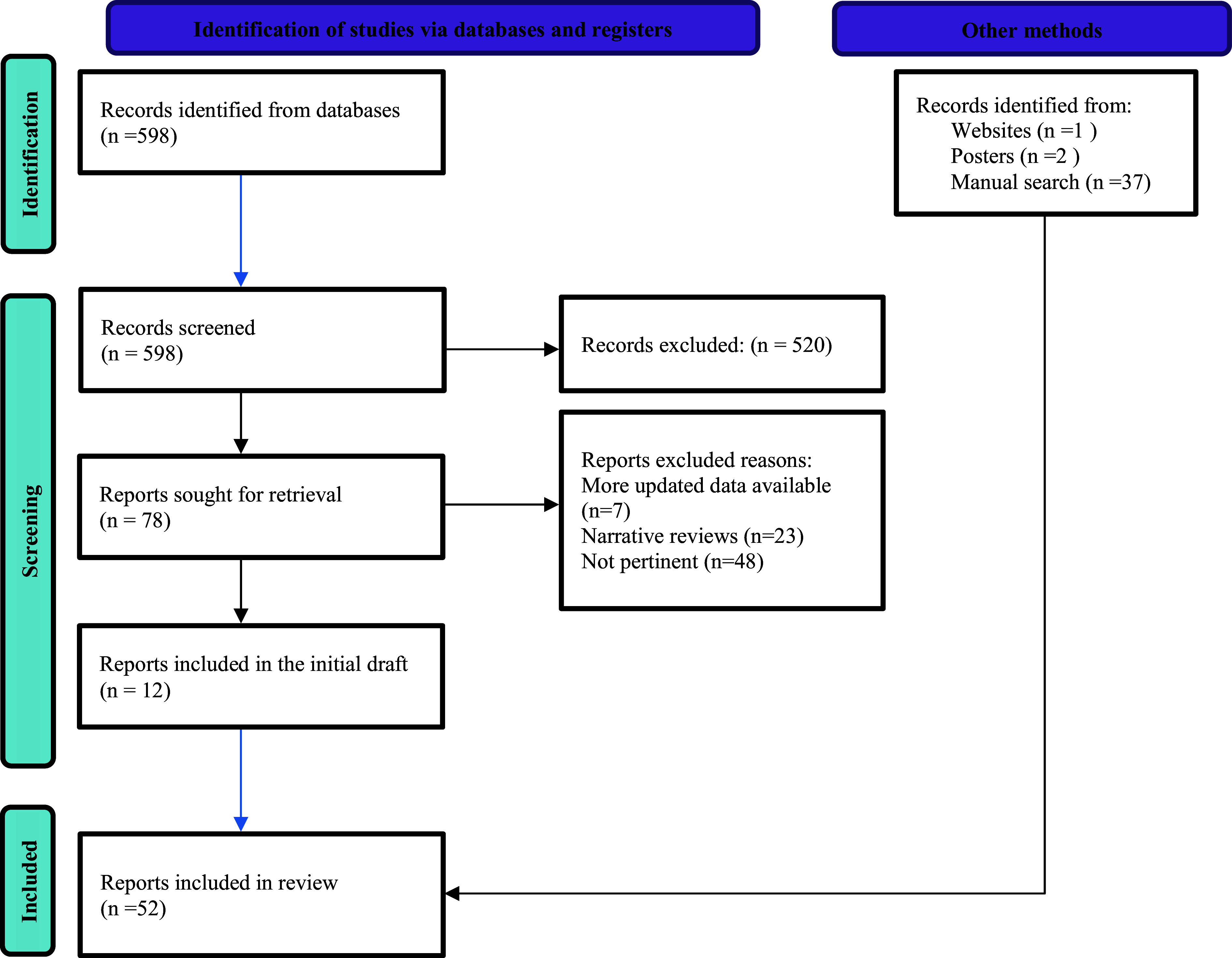
Search methodology.

The aim of this narrative review was to identify factors contributing to the diagnostic challenges of ASD, including the limitations of various screening and diagnostic tools. We searched for articles based on the diagnosis and screening of Autism Spectrum Disorder with specific attention to the tool Autism Diagnostic Observation Scale (ADOS), which is considered the gold standard. Our inclusion criteria were intentionally kept broad as we attempted to identify any article focused on diagnosis and screening for ASD. Our exclusion criteria were any unpublished material. Multiple databases were searched including PubMed, PsycINFO, Cochrane Library, Research Gate, Google, and Google Scholar. We looked for articles published in English with no geographical limitations. We included articles among all age groups including children and adults. We focused on the articles written over the last 10 years and concentrated on articles published after 2013, the year the DSM-5 was launched. The keywords and control vocabulary used include Autis* (Including Autism, Autism Spectrum Disorder, and Autistic Disorder), Asperger’s, Pervasive Developmental Disorder, Neurodevelopmental Disorder, Screening, Diagnosis, and ADOS. We also searched both manually and at PubMed Central to find relevant data. A Google search was used to find grey literature including data published as posters and on the CMS website. Individual studies are cited when needed to elaborate upon critical points. We found 598 articles that met the initial inclusion criteria and were screened. After reviewing their title and abstract, approximately 12 articles were used for the initial draft, with an additional 40 other articles were added later (Figure 1).

## Results

The emerging empirical literature highlights that ASD is frequently misdiagnosed or undiagnosed in certain populations, including females, individuals with higher cognitive functioning, and those from ethnic minority groups. This disparity arises in part because many screening and diagnostic tools were primarily validated on white male samples, leading to cultural and gender biases. Individuals with higher cognitive functioning, particularly females, often utilize compensatory strategies to mask social deficits, making accurate diagnosis more challenging. Additionally, diagnostic overshadowing—where comorbid conditions obscure ASD symptoms—further contributes to false negatives.

Although tools like the ADOS-2 are considered the standard for ASD diagnosis in controlled research settings, they have documented limitations. These include inconsistencies in validity when used in clinical practice, underscoring the need for more inclusive and robust diagnostic methodologies to enhance accuracy across diverse populations and settings.

## At-risk subgroups for misdiagnosis and the growing burden of undiagnosed ASD

Three distinct developmental phenotypes of ASD are likely to be missed or remain undiagnosed- females with ASD, individuals with ASD who possess higher cognitive abilities, and ethnic minority groups. The current diagnostic and screening measures, such as the Modified Checklist for Autism in Toddlers (M-CHAT), Autism Diagnostic Observation Scale (ADOS), and Autism Diagnostic Interview (ADI), have primarily been developed and validated utilizing samples that are mainly composed of males. This poses a significant challenge as these measures may lack sensitivity in detecting ASD in females, particularly those with higher cognitive and linguistic abilities.

To counteract gender bias, the second edition of the Autism Diagnostic Observation Scale (ADOS-2) has been revised to enhance its sensitivity in assessing females. A study aimed at examining the differential item and test functioning (DIF and DTF) of ADOS-2 items across sex and race revealed that, overall, there was no measurement bias across sex and race, except for hand mannerisms among female children and repetitive interests among African American children. In other words, ADOS-2 was reported to be equally accurate among males and females, indicating improved inclusivity in its application.^6^ These findings emphasize the need for ongoing efforts to develop and refine diagnostic tools that consider the diverse manifestations of ASD across different demographics in order to ensure accurate identification and support for all individuals irrespective of gender, cognitive abilities, and ethnic background.

Historically, ASD has shown higher prevalence in males, with estimates indicating a frequency 3–4 times higher among boys.^7^ This observed gender difference might be attributed to females exhibiting a tendency to compensate for the deficits associated with ASD. Early findings from a study examining the Toddler Module of the Autism Diagnostic Observation Scale-2 (ADOS-2) suggested that this diagnostic instrument measures social and communication milestones similarly for both male and female subjects. While the initial results suggested a lack of significant gender differences in how the ADOS-2 (Toddler Module) assesses social and communication milestones, the authors acknowledge potential confounding factors. Significantly, biases in the sample, developmental factors, or item selection could influence the observed results. The author emphasizes the importance of exercising caution and recognizes that these factors might obscure the interpretation of the results. Because of these confounds, the study highlights the importance of future follow-up research on the same sample. If subsequent investigations reveal gender differences over time, it could lend support to the notion of compensation in females with ASD.^8^ This study underscores the complexity of understanding ASD across genders and the ongoing efforts to refine diagnostic tools for accurate assessments.

Individuals with a high Intellectual Quotient (IQ) in the context of autism spectrum disorder (ASD) may exhibit a unique pattern of compensation for core deficits. For instance, they might effectively acquire social skills, mitigating challenges such as difficulty with eye contact or theory of mind. However, this compensation often comes at the cost of heightened levels of anxiety.^9^ The nuanced nature of their presentation implies that routine screenings and standard diagnostic tools may not adequately capture the complexity of ASD in individuals with a high IQ.

Cultural variations further contribute to the diversity in ASD symptoms. In Japan, for instance, cultural norms encourage children not to maintain direct eye contact with adults as a sign of respect. This cultural difference can pose challenges in diagnosing high-functioning individuals with ASD, as their behaviors align with cultural norms rather than being readily recognized as potential symptoms.^10^

Rural communities face unique challenges in accessing care for ASD. The Autism and Developmental Disabilities Monitoring (ADDM) data highlights significant disparities, with urban states recording evaluations at an earlier median age compared to rural states. Utah, Tennessee, and Missouri have the lowest percentage of evaluations recorded by the age of 36 months. This concerning underdiagnosis of ASD in rural United States emphasizes the need for targeted interventions and increased accessibility to diagnostic resources in these communities.^1^

Recent studies have identified significant limitations in the sensitivity and positive predictive value (PPV) of the Modified Checklist for Autism in Toddlers (MCHAT), the most used screening tool for autism spectrum disorder (ASD). These studies report sensitivity ranging from 33.1% to 38.8% and PPV from 14.6% to 17.8%.^11^ The findings suggest a need for a paradigm shift in ASD screening methodologies due to the current tools’ insufficient sensitivity and high rate of false negatives.

In a British population-based cohort study, researchers discovered that children living in poverty and those with higher intelligence often miss initial screenings.^12^ Consequently, it is crucial to focus ASD testing efforts on preschoolers from low-income families and minority groups. The employment of multilingual staff may enhance screening accuracy, given the MCHAT’s notably poor PPV among ethnic minorities. There exists a distinct and subtle ASD phenotype that lacks symptoms in the early stages, complicating detection before the age of four due to the heterogeneous nature of the disorder. These findings underscore the importance of longitudinal follow-up beyond 36 months.^2^

A significant disparity exists in ASD prevalence between states with urban populations (e.g., California and New Jersey) and those with rural populations (e.g., Missouri and Wisconsin). This disparity is likely attributable to changes in diagnostic criteria and higher healthcare literacy in urban areas. Additionally, state public health awareness programs play a crucial role in enhancing parental awareness of ASD.^2^ Addressing these multifaceted challenges is crucial for ensuring accurate and timely identification of ASD across diverse populations and settings.

## Enhancing diagnostics: strategies for improvement

ASD continues to be primarily diagnosed through clinical assessment, utilizing a range of screening and diagnostic tools to aid clinicians in the diagnostic process. Nevertheless, clinicians commonly exhibit reluctance to heavily rely on clinical judgment, a tendency often linked to an overemphasis on testing. This hesitancy to diagnose ASD clinically contributes to gaps in ASD-specific care, including challenges in conducting thorough suicidal risk assessments.^13^

Currently, there is no widely agreed-upon standardized approach or universally accepted standard of care for autism diagnosis. Different centers employ various screening and diagnostic tools (Table 1), including the Autism Diagnostic Observation Scale-2 (ADOS-2), Autism Diagnostic Interview-Revised (ADI-R), Childhood Autism Rating Scale (CARS), Screening Tool for Autism in Toddlers and Young Children (STAT), Modified Checklist for Autism in Toddlers (M-CHAT), Social Responsiveness Scale-second edition (SRS-2), Autism Spectrum Rating Scale (ASRS), Gilliam Autism Rating Scale (GARS), and others. The choice of tool often relies on the resources available and the clinician’s level of training.^3^

**Table 1.** Comparison between Diagnostic and Screening Tools for ASD

Tools	Age	Type	Sensitivity	Specificity	Settings
M-CHAT^14^	16–30 mo	Screening	0.97	0. 99	General
ASRS^15^	2–18 y	Screening	>0.90	>0.90	General
STAT^16^	2–3 y	Screening	0.92	0.85	Specialist
SCQ^17^	4+ y	Screening	0.75–0.85	0.60–0.75	General
SRS^18^	2.5	Screening	0.84–0.91	0.08–0.41	Specialist
GARS	3–22	Diagnosis	Evidence limited	Evidence limited	Specialist
ADI-R^19^	2+	Diagnosis	0.52	0.84	Specialist
ADOS^20^	2+	Diagnosis	0.94	0.80	Specialist
CARS^20^	2+	Diagnosis	0.80	0.88	Specialist

*ADOS:* Autism Diagnostic Observation Scale; *ADI-R:* Autism Diagnostic Interview-Revised; *CARS:* Childhood Autism Rating Scale; *STAT:* Screening Tool for Autism in Toddlers and Young Children; *MCHAT:* Modified Checklist for Autism in Toddlers; *SRS-2:* Social Responsiveness Scale-second edition; *ASRS:* Autism Spectrum Rating Scale; *GARS:* Gilliam Autism Rating Scale.

This variability in diagnostic tools underscores the need for ongoing research and standardization efforts within the field of ASD diagnosis. Establishing a consensus on best practices and enhancing clinician training can contribute to more accurate and consistent diagnoses, ultimately improving the quality of care for individuals with ASD.

The Autism Diagnostic Observation Scale-2 (ADOS-2) is often considered the gold standard for diagnosing autism spectrum disorder (ASD), demonstrating high sensitivity and specificity in research settings, ranging from .89 to .92 and .81 to .85, respectively.^21^ However, in clinical settings, while ADOS-2 maintains high sensitivity, specificity tends to be variable.^21^ Overall, ADOS-2’s sensitivity (0.94) and specificity (0.80) are comparable to other tools such as the Autism Diagnostic Interview-Revised (ADI-R) and Childhood Autism Rating Scale (CARS).^19^ Despite being the most sensitive among these tools, ADOS-2 exhibits modest specificity, leading to a higher likelihood of false positives.

The ADOS-2 comprises 5 modules tailored to the developmental and language capabilities of the individual being assessed. Module 1 is designed for children exhibiting minimal or no phrase speech, Module 2 is intended for those with phrase speech but not yet fluent, Module 3 is suitable for verbally fluent children and adolescents, and Module 4 is utilized for verbally fluent older adolescents and adults.

One study found an overall accuracy across modules of 70.4%, with a sensitivity of 90.9% and specificity of 66.0%. The accuracy decreases as the module number increases, with Modules 3 and 4 showing lower overall accuracy.^22^

The assessment of E-scores, which indicate emotional and behavioral issues in ADOS-2, includes symptoms like overactivity suggestive of attention deficit hyperactivity disorder (ADHD), disruptive behavior suggestive of mood disorders, and anxiety. These symptoms are common comorbidities, and research suggests that higher E-scores are associated with lower sensitivity and specificity.^23^ Therefore, in clinical samples characterized by a higher prevalence of comorbidity, ADOS-2 may not be as effective as a gold standard. A study administering ADOS-2 Modules 3 and 4 among patients aged 9–18 admitted to a child and adolescent psychiatric inpatient unit found lower sensitivity (Module 3: 58.3%; Module 4: 55.6%) and specificity (Module 3: 56.5%; Module 4: 59.5%) in this clinical context.^24^ This highlights the importance of considering the specific characteristics of the population being assessed when evaluating the effectiveness of diagnostic tools for ASD.

In a clinical sample of children and adolescents without autism spectrum disorder (ASD), a study reported a notable 34% false positives when ADOS-2 was administered. The identified group exhibited characteristics of high anxiety and low Restricted and Repetitive Behavior (RRB) symptoms. As a result, the study recommended against using ADOS-2 in isolation for ASD diagnosis and suggested incorporating structured parental interviews, which typically demonstrate better specificity. However, this approach has the potential to extend testing times, contributing to the existing issue of long wait times for assessments.^25^

In comparison to ADOS-2, other diagnostic tests for ASD are generally more cost-effective, quicker, easier to administer, and more readily available. However, these alternates are often underutilized. Despite their advantages, these tests exhibit significant variability in interrater reliability, influencing their sensitivity and specificity. Even ADOS-2, considered a more semi-structured test, demonstrates interrater reliability for diagnostic classification ranging between 64%–82%.^26^ It’s crucial to note that the published data on various tools often originate from research settings, and their performance in clinical samples may be lower. This emphasizes the need for ongoing research and efforts to improve the reliability and applicability of diagnostic tools for ASD in real-world clinical scenarios.

Various tests for autism spectrum disorder (ASD) exhibit variability in sensitivity and specificity, with clinical performance sometimes falling short of their research-derived values. In a recent meta-analysis focused on the M-CHAT-Revised with Follow-up (M-CHAT-R/F), the pooled sensitivity and specificity were notably high at 0.83 and 0.94, respectively, in screening for ASD among toddlers.^14^ Given that M-CHAT is one of the most readily available and free parent screening questionnaires for ASD in toddlers, these results are promising. However, it’s important to note that, like other tools, translated versions of M-CHAT in different languages often exhibit lower sensitivity and specificity.

Diagnosing ASD can be particularly challenging, especially for individuals with high levels of functioning and comorbid mental illnesses, who are more likely to yield false negatives in testing. In such cases, a structured interview and a comprehensive assessment of comorbidities can be valuable tools to support the clinical diagnosis of ASD, especially when suspicion is high.

## Overshadowed clues: clinical screening strategies

There is a wide range of distinct comorbid conditions closely associated with individuals with milder symptoms of ASD, often overshadowing core symptoms.^27^ With the ontogenic unfolding of complex human development, widespread variability in the onset and heterotopic continuity of these symptoms is observed during its different stages. Firstly, in infancy and toddlerhood, the subtle signs and symptoms of ASD are often overlooked as normative, even though speech delays, restrictive interests, atypical sleep patterns, and sensory processing problems could be present at these stages.^28^ Many factors not limited to misinterpretation of screening tools, access to mental health, clinical training, clinician’s bias, and stigma contribute to missed diagnostics.

In preschoolers and school-age groups, ADHD remains a more frequent diagnosis among ASD youths, increasing the burden of undiagnosed ASD phenotypes even though there are suboptimal responses to first-line stimulants. Until 2015, before the inception of DSM-5, ASD, and ADHD were mutually exclusive diagnoses, usually leading to an overemphasis on ADHD due to the stigma and clinicians’ hesitancy to diagnose ASD. With widespread misinformation suggesting neuropsychological testing tools are required for ASD diagnosis, clinicians’ hesitancy is often amplified by policies (managed care, school, etc.) that are not supported by empirical evidence.^29^

In late school-age children, social anxiety disorder frequently overshadows ASD diagnoses due to lagging social skills,^30^ and likewise, obsessive-compulsive disorder due to restrictive interests being misinterpreted as compulsions.^31^ If an ASD diagnosis is missed until adolescence, individuals struggling with social skills often camouflage their symptoms while feeling pressured to fit in.^32^ These myriad clinical presentations in early adolescence are complicated by self-harm, often mimicking symptoms of borderline personality disorder.^33^ Though self-harm is contextual and phenomenologically different in ASD, it is frequently misunderstood and overshadowed by BPD. It’s not uncommon for ASD individuals to continue to have BPD diagnosis during transitional age years until adulthood.^34^

Similarly, psychotic symptoms are not uncommon in ASD, potentially leading to serious adulthood trajectories that resemble schizophrenia, even though first-rank symptoms are absent. The notable rise in ASD diagnoses, partly due to the inclusion of milder forms has led to challenges in distinguishing ASD from psychotic disorders (PD). Individuals with ASD may show ‘pseudopsychotic’ symptoms like those of psychosis. As a result, ASD is often misdiagnosed as PD. Conversely, new psychotic disorders in those with ASD can be missed because the core symptoms of autism overshadow them. Studies report that the rate of PD in adults with ASD is at least 10 times higher than in the general population, underscoring the importance of accurate diagnosis.^35^

In recent years, Avoidant/restrictive food intake disorder has been frequently associated with ASD^36^; however, emerging evidence suggests that adults with Anorexia Nervosa score higher on the ADOS-2 algorithms.^37^ Restrictive eating behaviors should be carefully evaluated as a potential indicator of ASD, particularly in adults with minimal observable impairments.^38^

This paradigm underscores the importance of detailed clinical assessments and contextual correlation to ensure accurate diagnoses for individuals with ASD, particularly those with milder subtle symptoms as often cooccurring disorders overshadow its early identification.

It is important to note that autism can also be confused with bipolar disorder^40^ and borderline personality disorder,^33^ both of which manifest traits resembling autism and ruminative thinking. In many cases, individuals on the spectrum are diagnosed with bipolar disorder, especially when they experience heightened activation or irritability on SSRI. Aside from improving access to diagnostic and screening tools, there is a need for clinician education and improvement in comorbidity assessment.^39^

ASD is frequently overlooked, underdiagnosed, and sometimes misdiagnosed, influenced by various contributing factors. Initial signs and symptoms are frequently perceived as normative development patterns in many instances, leading to a reluctance for reevaluation when improvement is not observed after a period of watchful waiting.^41^ Symptoms such as increased irritability heightened susceptibility to anger, poor eye contact, communication delays, and the presence of sensory issues manifest across a spectrum of variable intensity in the dimensional realm of ASD symptoms.

A thorough examination often necessitates a detailed developmental history, including inquiries about potential complications during and after pregnancy. Concerns related to frequent crying, difficulties with gastrointestinal symptoms, poor eye contact, language developmental challenges, struggles in forming friendships, and notable aggression during childcare interactions, may raise suspicions warranting ASD screening. While the M-CHAT tool is integrated into pediatric clinics and child development assessments, it may overlook subtle signs crucial for ASD identification. Recognizing this limitation, various software-based face and voice recognition applications are under development.^42^ Given the elusive nature of these subtle symptoms for such tools, direct questioning remains a clinically sound approach. Additionally, exploring alternative biomarkers through unconventional technology could offer valuable insights beyond conventional measures.

In clinical settings, a distinctive developmental history unfolds among children diagnosed with ASD. These children encounter challenges in forming friendships with peers of their age and frequently exhibit a strong inclination toward technology, particularly video games, and a keen interest in music, puzzles, games, collectible cards, dinosaurs, and Lego sets. Notably, reports indicate that children on the spectrum often show an affinity for Japanese culture, particularly through an understanding of anime, and they display curiosity about different languages.

Children with ASD also commonly experience sensory issues, clinically manifested as heightened sensitivity to various textures, colors, and smells. They may resist activities such as haircuts and prolonged showers, and exhibit aversions to tight clothing or touch-related sensory stimuli, often cutting tags from their T-shirts.^43^ Additionally, these children often have comfort items and unique interests, such as a fascination with marine biology, and forensic science, a penchant for horror movies and crime thrillers, a specific liking for certain types of food, and difficulties in engaging in reciprocal peer interactions.

Many of these symptoms may not become apparent until the child reaches the 5th to 6th grade, typically between 11 and 12 years old, when distinct gender differences emerge. Females on the spectrum often display more eye contact and employ compensatory behaviors, including language use and camouflaging. The hypothesis posits that the lack of reciprocal feedback from peers, coupled with challenges in interacting with same-age peers, and the cognitive exhaustion from constant camouflaging to fit in, can contribute to mental health crises in this population.^44^

**Figure 2.**
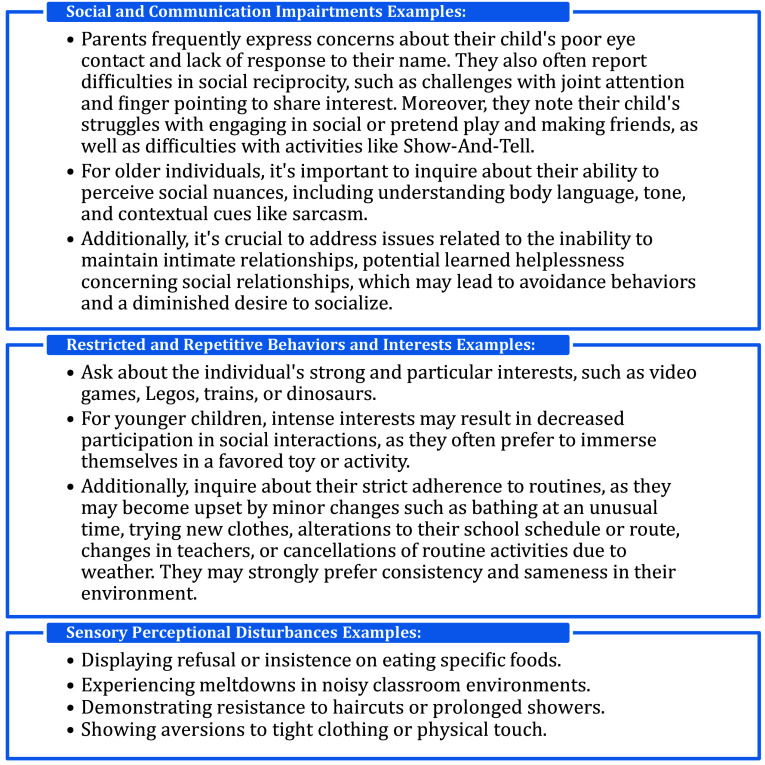
How to ask narrow questions to screen for ASD in complex phenotypic presentation.

Children and adolescents with ASD, initially presenting in mental health settings, may exhibit symptoms resembling social anxiety disorder or generalized anxiety disorder. However, their challenges stem from deficits in theory of mind, shaping their unique clinical phenotypes, rather than a fear of negative scrutiny.^33^ Non-suicidal self-injury is not uncommon among this demographic, and they may be mistakenly diagnosed with borderline personality disorder due to defiance and interpersonal difficulties, despite the absence of chronic feelings of emptiness.^45^ It’s crucial to recognize that ASD typically emerges at an earlier age, often showing symptoms around three to 4 years old. In contrast, higher-functioning ASD symptoms may become apparent later, during school age and pre-puberty. Recent empirical data indicate that borderline personality disorder tends to manifest around the age of 11 or 12, and a DSM-5 diagnosis can be made if symptoms persist consistently over 1 year. Additionally, some children with ASD may be misdiagnosed with serious and enduring mental health disorders like schizophrenia or intellectual disability, as psychotic symptoms are not uncommon in this population (Figure 2).^27^

Therefore, a thorough chronological assessment of symptom ontology, considering distinct symptom clusters emerging at an early age, deficits in theory of mind, sensory issues, social challenges, a preference for routine, and atypical language development, is essential for accurate diagnostics (Tables 2 and 3).

**Table 2.** A Symptom Checklist Based on Domains of Impairments in Individuals with ASD

Domains of development
Physical: Delay in physical milestones is common but not characteristic of ASD
Cognitive: Cognitive delays, although common, are not a characteristic of ASD
Language: Although language issues themselves do not suggest ASD, language and communication delays are common in ASD
Social: Social impairments are characteristic of ASD
*Autism is characterized by its 2 core symptoms of Social and Communication Impairment along with Restricted and Repetitive Interests and Behaviors. Comorbidities like Sensory Perceptional Disturbances are associated with ASD but are not characteristic of ASD.*

Social and communication impairments
Impairment is more pronounced among individuals who are non-verbal or have limited verbal skills or delays in communication.
Nonetheless, even individuals with typical language abilities encounter challenges in understanding the subtleties of social interactions, such as social reciprocity difficulties, trouble making friends, and poor eye contact.
In younger children, assessing their capacity for social play is crucial, as it involves navigating complex interactions, such as taking turns, grasping contextual cues, and advancing the storyline. Children on the autism spectrum often struggle with social reciprocity and comprehending the intricacies of social interactions.
Older individuals may find it challenging to form intimate relationships despite previous attempts to learn, leading to avoidance and a lack of interest in socializing. They may struggle to interpret sarcasm, body language, and tone, which can impact their relationships, despite succeeding in their professional lives.
Gender differences exist, with females often exhibiting improved behaviors such as increased eye contact, suggesting greater compensatory mechanisms.

Restricted and repetitive behaviors and interests (RRBI)
Previously referred to as stereotypies, individuals with autism commonly exhibit intense, limited, and restricted interests.
They often display a strong preference for technology, such as video games, music, puzzles, dinosaurs, legos, and trains.
They adhere strictly to routines and may become upset by any changes, insisting on the sameness of their environment.
In younger children, intense interests are evident as they may show little desire for social interactions, instead immersing themselves in a preferred toy or activity.
Individuals with autism may become upset by minor changes, such as alterations to their school route, different teachers for a particular subject, or being unable to play at the scheduled time due to weather conditions.
Older individuals may exhibit unique interests, which may include a fascination with complex sciences like marine biology, forensics, or horror movies.

Sensory perceptional disturbances (SPD)
While sensory perception disturbances are not inherently characteristic of autism, they are notably more prevalent among individuals on the autism spectrum.
Parents often begin to suspect autism in their children when they observe significant sensory issues, such as heightened sensitivity to various textures, touch, or smells.
A thorough investigation into the individual’s hypo- or hypersensitivity to the five senses, as well as proprioception, can be immensely beneficial in understanding certain behaviors.
At times, these behaviors can be explained by the need to avoid stimuli to which they are hypersensitive or by sensory-seeking behavior. For instance, they may resist haircuts and extended showers or exhibit aversions to tight clothing or touch-related sensory stimuli.

Comorbidities
Developmental background: Investigate possible prenatal complications and any delays in development.
Gastrointestinal symptoms: Common signs include frequent crying, colic-like behavior, and recurring GI issues.
Anxiety: Children with autism may sometimes be mistaken for those with social anxiety disorder. Their insistence on sameness can be misinterpreted as obsessive-compulsive disorder.
Mood instability: Aggressive behavior during social situations may be notable.

**Table 3.** Effective Strategies for Incorporating Empirical Data Gaps into Clinical Practice and Decision-Making.

Critical question	Empirical evidence	Clinical significance
ASD prevalence^1^	The prevalence of autism spectrum disorder (ASD) is estimated to be 1 in 36 among children aged 8.	The increased prevalence is not unexpected given the growing clinical burden of undiagnosed ASD.
ASD prevalence in psychiatrically referred population^4^	Studies indicate that ASD prevalence is 5 times higher in psychiatrically referred populations compared to community samples.	Universal screening for ASD, particularly in settings like inpatient facilities, is essential.
Mis/undiagnosed^6^ ^,^^7^ ^,^^8^ ^,^^9^ ^,^^10^	Research identifies three distinct phenotypes of mis/undiscovered ASD cases.	Screening tools like MCHAT, ADOS, and ADI have been developed and validated primarily in males.
Is male preponderance attributed to ADOS–2?^7^ ^,^^8^	Gender differences are not evident in the ADOS–2 Toddler Module.	Ongoing modifications to ADOS–2 aim to capture gender differences, supported by recent studies indicating no significant variance.
How do ASD individuals with higher IQs differ clinically?^9^	Individuals with ASD and higher IQ tend to exhibit a greater degree of compensatory abilities.	High-functioning individuals, particularly females, may test negative and consequently remain undiagnosed.
Is there a rural vs Urban divide?^1^ ^,^^2^	Rural areas show lower rates of ASD evaluations recorded by the age of 36 months.	Enhanced accessibility to diagnostic resources is crucial in rural communities to ensure timely diagnosis and intervention.
How do ADOS–2 modules vary?^22^	The accuracy of ADOS–2 modules decreases with the age of the individual.	ADOS–2 demonstrates reduced accuracy in diagnosing ASD in older individuals.
Why may ADOS–2 yield false negatives?^24^	ADOS–2 exhibits low specificity in clinical samples, leading to a higher likelihood of false negatives.	High-functioning individuals are particularly susceptible to false negatives when assessed using ADOS–2.
How do E scores affect sensitivity and specificity?^23^	Higher E-scores on ADOS–2 are associated with decreased sensitivity and specificity.	ADOS–2 tends to be less accurate in individuals with emotional and behavioral disturbances, reflected in higher E-scores.
Why should ADI be added to ADOS–2?^25^	ADOS–2, with its low specificity, may yield false negatives. Supplementing it with ADI, which offers higher specificity, can enhance diagnostic accuracy.	Combining ADOS–2 with ADI-R results in more precise diagnostic outcomes.
What’s the significance of ADOS–2 interrater reliability?^26^	The interrater reliability of ADOS–2 for diagnostic classification ranges from 64% to 82%.	Interrater reliability plays a crucial role in determining the sensitivity and specificity of ADOS–2 in diagnosing ASD.
Does setting affect ADOS–2 performance?^21^ ^,^^24^	ADOS–2 demonstrates high sensitivity and specificity in research settings, but exhibits variable specificity in clinical settings.	The optimal use of ADOS–2 is validated for outpatient-based clinical settings, performing sub-optimally in other contexts like inpatient settings.
Who is at risk of false positives with ADOS–2?^25^	Studies report a substantial 34% rate of false positives when utilizing ADOS–2.	Individuals showing characteristics of high anxiety and low Restricted and Repetitive Behavior (RRB) symptoms are prone to false positives when assessed using ADOS–2.

## The clinical ramifications of increasing burden

The efficacy of treatment for individuals with ASD relies heavily on early accurate diagnostics. When co-occurring conditions are treated without considering the underlying ASD, responses can be suboptimal, with potential paradoxical reactions or outcomes.^27^ Children and adolescents with ASD may have subdued responses when treated with serotonin reuptake inhibitors or stimulant medications without recognizing the additional diagnosis of ASD.^46^ ^,^^47^ The lack of integration with multi-modal interventions such as social skills training and speech therapy can lead to increased morbidity and poor overall outcomes. When conditions become refractory to these treatments, it places additional strain on caregivers and fosters a negative view of mental health among patients and families.^48^ Therefore, recognizing and accurately diagnosing ASD before initiating treatment is critical. Many treatments require appropriate modifications, including the use of therapeutics and the addition of multi-modal interventions. The modifications while considering medication dosage, psychological interventions, and the treatment of other co-occurring mental health and medical conditions are essential to achieving the desired outcomes.^39^

Early diagnosis of autism spectrum disorder (ASD) is crucial to provide timely and appropriate care. Applied Behavior Analysis (ABA) therapy is considered evidence-based for severe symptoms, but its cost-effectiveness remains a concern. A study estimates that around 37% of individuals with ASD do not receive any therapy, and 30% do not receive any treatment at all.^49^ Some individuals may be unaware of their ASD condition, and this lack of support is alarming, especially in light of the association between ASD and other mental health conditions such as depression, anxiety, and suicidality.^50^ ^,^^51^ A study conducted in the New York University Pediatric Psychiatry Emergency Room found that less than half of clinicians believe autism is an independent risk factor for suicide.^13^

Medication use, including polypharmacy, is widespread in the ASD population. However, there is a diverse response to medications, mirroring the heterogeneity of autism itself. Selective Serotonin Reuptake Inhibitors (SSRIs), commonly used for mental health conditions, may not be as effective in individuals with ASD and could potentially lead to harm due to behavioral activation.^52^ Stimulants may not exhibit a linear dose–response curve in individuals with ASD.^29^ Given the sensory-perceptual disturbances associated with autism, individuals with ASD are more susceptible to side effects from medications. It’s noteworthy that almost all medications used in ASD, except for aripiprazole and risperidone, are considered off-label.^39^ Addressing the challenges in providing appropriate therapies and medications for individuals with ASD is crucial for improving their overall well-being and mental health outcomes.

## Conclusion

Enhancing early identification of ASD requires developing precise diagnostic tools tailored to specific demographics, such as females, higher cognitive abilities individuals, and ethnic minorities. Adequate funding for diagnostic tests and therapeutics is crucial to support timely interventions. Improving service accessibility, especially for rural and lower socioeconomic areas, is essential to reduce disparities. Training healthcare providers to confidently diagnose and refer ASD cases can decrease wait times, while education on assessing comorbidities can ensure accurate diagnoses. A multi-faceted approach involving targeted diagnostics, increased funding, improved accessibility, and enhanced provider training is necessary for timely and appropriate ASD care.

## Data Availability

No datasets were generated or analyzed during the current study.

## Abbreviations

ASD: Autism Spectrum Disorder
ER: Emergency Room
ADDM: Autism and Developmental Disability Monitoring Network
CMS: The Centers for Medicare & Medicaid Services
DIF: Differential Item Functioning
DTF: Differential Test Functioning
IQ: Intellectual Quotient
RRB: Restricted and Repetitive Behaviors
SSRI: Selective Serotonin Reuptake Inhibitors
DSM-5: Diagnostic and Statistical Manual Fifth Edition
ABA: Applied Behavioral Analysis.
ADOS: Autism Diagnostic Observation Scale
ADI-R: Autism Diagnostic Interview-Revised
CARS: Childhood Autism Rating Scale,
STAT: Screening Tool for Autism in Toddlers and Young Children
MCHAT: Modified Checklist for Autism in Toddlers
SRS-2: Social Responsiveness Scale-second edition
ASRS: Autism Spectrum Rating Scale
GARS: Gilliam Autism Rating Scale
